# Wound Administration of M2-Polarized Macrophages Does Not Improve Murine Cutaneous Healing Responses

**DOI:** 10.1371/journal.pone.0102994

**Published:** 2014-07-28

**Authors:** Nadine Jetten, Nadia Roumans, Marion J. Gijbels, Andrea Romano, Mark J. Post, Menno P. J. de Winther, Rene R. W. J. van der Hulst, Sofia Xanthoulea

**Affiliations:** 1 Department of Molecular Genetics, CARIM, Cardiovascular Research Institute Maastricht, Maastricht University, Maastricht, The Netherlands; 2 Department of Plastic Surgery, NUTRIM, School for Nutrition, Toxicology & Metabolism, Maastricht University Medical Centre, Maastricht, The Netherlands; 3 Department of Pathology, CARIM, Cardiovascular Research Institute Maastricht, Maastricht University, Maastricht, The Netherlands; 4 Department of Gynecology, GROW, School for Oncology and Developmental Biology, Maastricht University Medical Centre, Maastricht, The Netherlands; 5 Department of Physiology, CARIM, Cardiovascular Research Institute Maastricht, Maastricht University, Maastricht, The Netherlands; University Hospital Hamburg-Eppendorf, Germany

## Abstract

Macrophages play a crucial role in all stages of cutaneous wound healing responses and dysregulation of macrophage function can result in derailed wound repair. The phenotype of macrophages is influenced by the wound microenvironment and evolves during healing from a more pro-inflammatory (M1) profile in early stages, to a less inflammatory pro-healing (M2) phenotype in later stages of repair. The aim of the current study was to investigate the potential of exogenous administration of M2 macrophages to promote wound healing in an experimental mouse model of cutaneous injury. Bone marrow derived macrophages were stimulated in-vitro with IL-4 or IL-10 to obtain two different subsets of M2-polarized cells, M2a or M2c respectively. Polarized macrophages were injected into full-thickness excisional skin wounds of either C57BL/6 or diabetic db/db mice. Control groups were injected with non-polarized (M0) macrophages or saline. Our data indicate that despite M2 macrophages exhibit an anti-inflammatory phenotype in-vitro, they do not improve wound closure in wild type mice while they delay healing in diabetic mice. Examination of wounds on day 15 post-injury indicated delayed re-epithelialization and persistence of neutrophils in M2 macrophage treated diabetic wounds. Therefore, topical application of ex-vivo generated M2 macrophages is not beneficial and contraindicated for cell therapy of skin wounds.

## Introduction

Cutaneous wound healing is a complex and highly dynamic process in which the interaction between resident cells of the skin, inflammatory leukocytes, extracellular matrix components and soluble mediators is essential. Inflammation and inflammatory cells play a crucial, although still not completely defined, role during cutaneous healing responses. They exert effector functions such as immune defence against invading pathogens, but they are also involved in tissue repair responses by, among other mechanisms, stimulating growth factor and anti-inflammatory cytokine production that are necessary for resolution of inflammation and repair. However, excessive inflammation during the wound healing process can result in derailed tissue repair like is the formation of ulcers, hypertrophic scars, keloids and in chronic wounds [1], [2].

Macrophages at the wound site consist of two populations of cells: the resident tissue macrophages at a relatively low density of 1–2 cells per mm^2^ [3], and the newly recruited cells from either circulatory monocytes or splenic reservoirs that differentiate in response to tissue injury [4]. Depletion of resident skin macrophages (but not the inflammatory monocyte subset), with an anti-CSF1R antibody, was shown to have no major effect in a macrophage-dependent epithelial damage wound healing model in mice [5]. Conversely, several studies have shown that total depletion of macrophages during wound healing has a detrimental effect on repair with defective wound re-epithelialization, angiogenesis, granulation tissue formation and contraction and with increased pro-inflammatory cytokine and reduced growth factor expression. Moreover, these studies have delineated that macrophages exhibit different functional phenotypes during the different phases of repair [6]–[8].

Macrophages have the ability to exhibit a variety of phenotypic profiles in response to the microenvironmental stimuli to which they are exposed. In a simplified version, they polarize towards either a pro-inflammatory microbicidal cell type, upon stimulation with microbial agents e.g. LPS and/or IFNγ (the classically activated or M1 macrophage) or to an alternative activated phenotype characterized as anti-inflammatory (M2 macrophage). Based on their functionality and expression of surface markers, M2 macrophages can be further subdivided into subsets: M2a, activated by IL-4 or IL-13; M2b, activated by immune complexes and agonists of Toll-like receptors (TLRs) or IL-1R; M2c, activated by IL-10 and glucocorticoids [9], [10].

Studies performed in murine wounds showed that wound macrophages exhibit a complex phenotype that includes both classically and alternative activation traits and their polarization profile changes as the wound matures [11]. In addition, several studies have determined a causative role of a dysregulation of macrophage polarization in different types of chronic non-healing wounds. It was shown that persistence of an unrestrained M1 macrophage population with incomplete switch to an anti-inflammatory, pro-healing M2 phenotype is a common feature of non-healing wounds such as chronic venous leg ulcers [12] or diabetic wounds of humans and mice [13]–[15]. From these and other studies [16], TNF appears to be a key effector molecule released from the derailed macrophages and its inhibition results in significant improvement of healing.

Since adoptive transfer of polarized macrophages was shown to reduce severity of different inflammatory diseases in mice[17]–[21], we investigated the hypothesis that exogenous administration to murine wounds of in-vitro generated M2 macrophages, during the inflammatory phase of healing, might shift the wound environment towards a more anti-inflammatory, pro-healing profile and accelerate wound repair.

## Materials and Methods

### 2.1. Animals and wound model

C57BL/6 (10 to 12 weeks), Lepr^db^ (10 weeks) female mice (Charles River) or ROSA26-EGFP transgenic mice [22] (a generous gift from Prof. Rene H. Tolba; RWTH Aachen) were used for experiments. To generate wounds, mice were anesthetized by isofluorane inhalation and the dorsal surface was shaved and cleaned with 70% ethanol. Full-thickness excisional skin wounds were generated on either side of the dorsal midline using a 4 mm biopsy punch (Kai medical, Tokyo, Japan). Wounds were separated by a minimum of 5 mm of uninjured skin and both wounds were injected with the same solution to avoid the possibility that injected cells could migrate or secrete substances that could influence contralateral wound. Mice were housed individually in special paper bedding material (7089 Harlan Teklad Diamond Soft Bedding), to avoid bedding particles interfering with the healing wounds. Two or four wounds were generated on the same animal and macrophages (0.5x10^6^ cells) or control solutions (RPMI without supplements added, referred to as saline) were injected with three injections of 20 µl around the wounds after 1 and 3 days from wounding. At sacrifice, wounds were excised and snap frozen in liquid N_2_ or processed for generation of paraffin or cryosections. Mice were maintained under standard pathogen-free conditions and all experiments were approved by the Committee for Animal Welfare of Maastricht University. The investigation conforms to the *Guide for the Care and Use of Laboratory Animals* published by the US National Institutes of Health (NIH Publication No. 85–23, revised 1996).

### 2.2. Bone marrow isolation and macrophage polarization

For the generation of bone marrow derived macrophages (BMDM), bone marrow was isolated from femur and tibia bones of db/+ mice and cultured in 15-cm bacteriologic plastic petri dishes in RPMI 1640 supplemented with 10% heat inactivated fetal bovine serum, 100 U/ml penicillin, 100 µg/ml streptomycin, 2 mM L-glutamine, 10 mM Hepes (all Gibco, Invitrogen) and 15% L-929 cell conditioned medium (LCM) containing M-CSF for 8 days. After 8 days, differentiated macrophages were harvested and plated overnight in either 6-well (2x10^6^ cell/well) or 24-well (0.4x10^6^ cells/well) plates. The following day, cells were polarized for 24 h with either IL-4 (20 ng/ml, Peprotech) for M2a or IL-10 (10 ng/ml, R&D Systems) for M2c macrophages. Non-polarized cells are referred to as M0 macrophages. For in-vivo injections, cells were harvested after 24 h of polarization, counted and resuspended in RPMI medium alone (without supplements added). For quantification of gene-expression, cells were either harvested after 24 h of polarization for RNA isolation or were washed and stimulated for an additional 24 h with LPS (100 ng/ml, L2630 Sigma-Aldrich), after which RNA was isolated.

### 2.3. RNA isolation and quantitative gene expression

RNA was isolated either with the RNeasy Fibrous Tissue kit (Qiagen GmbH, Hilden, Germany) for mouse wound tissue or with Trizol (Sigma-Aldrich Chemie BV) for cell monolayers. Residual DNA was digested with the RQ1 RNase-free DNase (Promega GmbH, Mannheim, Germany) and cDNA synthesis was performed using the iScript cDNA synthesis kit (Bio-Rad, Hercules, CA, USA). Quantitative real-time PCR was performed in 10 ng of cDNA, with 1×Absolute qPCR SYBR Green Fluorescein Mix (Westburg, Leusden, The Netherlands) and 150 nM of gene specific forward and reverse primers. Cyclophilin A and β-actin were used as housekeeping genes. Primer sequences are indicated in Table S1.

### 2.4. Immunohistochemistry and immunofluoresence

For immunofluorescent detection of eGFP macrophages, wounds were isolated and fixed in 4% paraformaldehyde with 10% sucrose in PBS for 6 h. Fixed wounds were bisected in caudocranial direction and embedded in O.C.T. Tissue tek (Sakura Finetek) with cut surface facing down. Frozen sections (7 µm) were fixed for 5 min. in acetone, washed with PBS and counterstained with 4′,6-diamino-2-phenyl indole (DAPI, Molecular Probes). For immunohistochemical detection, excised wounds were bisected in caudocranial direction and embedded in OCT compound or fixed in formalin. Paraffin sections (4 µm) were stained with haematoxylin and eosin (H&E), Martius-scarlet-blue trichrome (MSB) or picrosirius red. Frozen sections (7 µm) were stained for macrophages with rat anti-mouse F4/80 (AbD Serotec), for endothelial cells with rat anti-mouse CD-31 (BD Pharmingen), and for neutrophils with rat anti-mouse NIMP (BD Pharmingen) and counterstained with haematoxylin.

### 2.5. Morphometric analysis

Wounds were photographed every two days and wound area was quantified using Image J. The percentage of wound closure was calculated using the following formula: wound closure (%)  =  [(wound area on day 0 – wound area on indicated day)/wound area on day 0] x 100. The extent of re-epithelialization was determined on H&E paraffin tissue sections. Photographs of wound sections were used to measure the distance between the tips of the migrating epithelial tongues, by ImageJ software. Organization and maturation of collagen bundles was assessed on paraffin sections of wounds stained with picrosirius red and analyzed by polarized light microscopy. Macrophages, neutrophils, endothelial cells and fibrin/erythrocytes in MSB staining were determined by quantifying the red-stained area in three representative rectangles in the granulation tissue at a 200x magnification (HPF), including the area below the scab (excluding scab) and areas left and right of it. The area of macrophages, neutrophils, endothelial cells or fibrin/erythrocytes for each high power field (HPF) was measured by Photoshop CS2. Staining results were equivalent to the semi-quantitative microscopic analyses of the wounds for these parameters on H&E stained slides by an experienced pathologist (M.J.G.).

### 2.6. Statistical analysis

Statistical analyses were performed using Graphpad Prism or SigmaPlot. Analyses compared each measurement with the relative control under the assumption that the variables analyzed are independent and significance of difference was analyzed using unpaired Student t-test. Data are expressed as means ± SEM. A p≤0.05 is considered statistically significant.

## Results

### 3.1. Exogenously administered macrophages localize in wounds for several days

**Figure 1:**
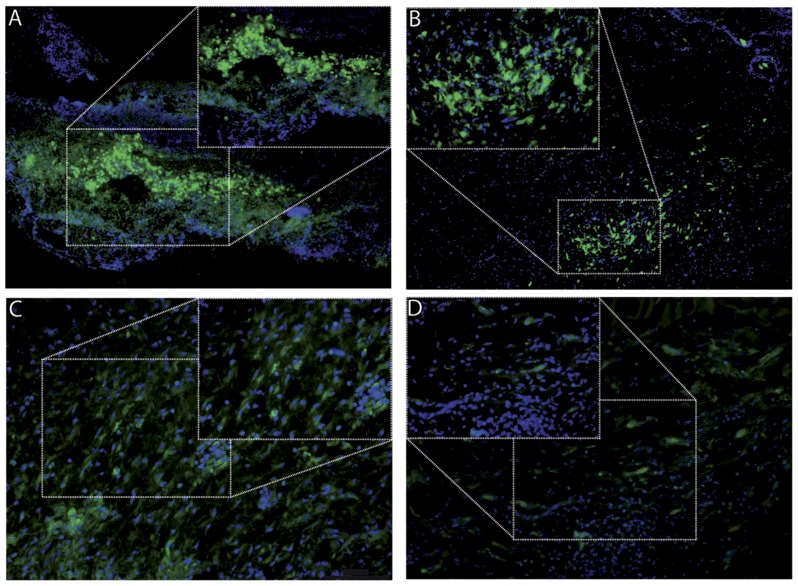
Visualization of eGFP macrophages in wound tissue. eGFP expressing macrophages were injected in full-thickness excisional skin wounds and tissue was isolated (A) 3 days, (B) 4 days, (C) 6 days and (D) 8 days after injection, and visualized for eGFP expression by fluorescence microscopy.

To determine the fate of exogenously injected macrophages into skin wounds, bone marrow derived macrophages were differentiated from ROSA26-EGFP transgenic mice [22] and 0.5x10^6^ cells were injected intradermally around the wound at three injection sites one day after wounding. Wounds were isolated 3, 4, 6 and 8 days later and analyzed for the presence and localization of eGFP expressing macrophages. As shown in Fig. 1, macrophages are clearly present in the wound bed 3, 4 and 6 days after injection and they persist, albeit in lower numbers and exhibiting less intense fluorescence, 8 days after injection.

### 3.2. Generation of M2a and M2c macrophage populations

**Figure 2:**
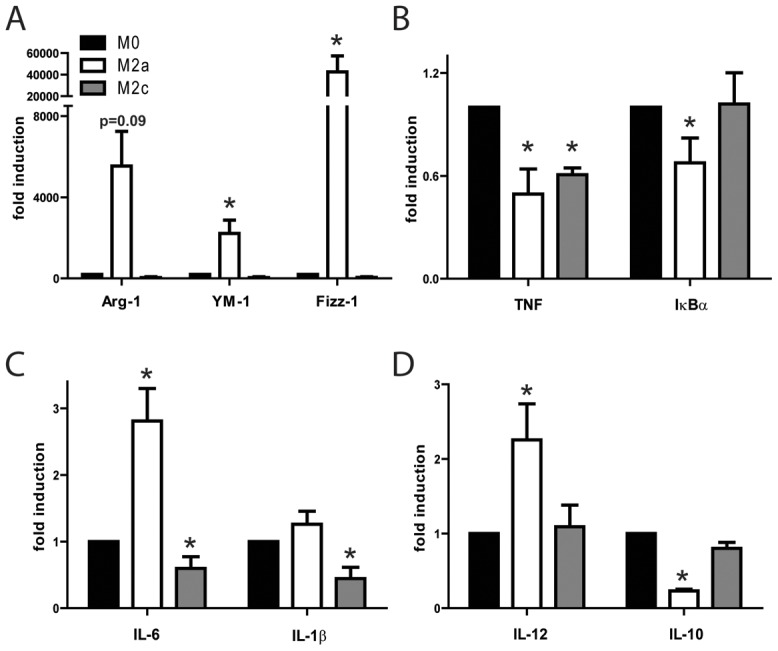
Induction of M2 macrophages. Phenotypic characterization of polarized macrophages. (A) Gene expression analysis for M2 markers in M0 control, M2a and M2c bone marrow derived macrophages (BMDM) after 24 h of IL-4 (for M2a) or IL-10 (for M2c) polarization. (B-D) Gene expression analysis for inflammatory markers in M0 control, M2a and M2c polarized cells after 24 h of polarization and an additional 24 h of LPS stimulation. Cells were analyzed in triplicates and the mean expression of 2–3 independent experiments is indicated. M0 control levels were arbitrarily set to 1 in each experiment and for each gene and statistical significance was evaluated analyzing M2a and M2c expression levels compared to M0, using a one-sample t-test analysis against the hypothetical value 1 (M0). *p≤0.05.

To study the effect of the exogenous administration of M2-polarized macrophages on cutaneous wound healing responses, bone marrow derived macrophages were generated and polarized for 24 h with either IL-4 (20 ng/ml) or with IL-10 (10 ng/ml) in order to generate two distinct M2 macrophage subsets, M2a and M2c respectively [10]. A parallel set of cells was further stimulated for an additional 24 h with LPS, in order to study the resulting inflammatory profile. Gene expression analysis confirmed correct macrophage polarization, with M2a cells expressing high levels of M2a markers like Arginase-1, YM-1 and Fizz-1 (Fig. 2A). Although, as previously shown [10], similar polarization markers are not so clearly present in M2c macrophages, cytokine expression analysis indicated that these cells exhibit an alternative polarization profile compared to non-polarized M0 macrophages, with reduced expression of pro-inflammatory cytokines like TNF, IL-6 and IL-1β. Interestingly, M2a cells exhibited an intermediate inflammatory profile with reduced expression of pro-inflammatory genes like TNF and IκBα, but also increased pro-inflammatory IL-6 and IL-12 and reduced anti-inflammatory IL-10 expression (Fig. 2B-D).

### 3.3. M2-polarized macrophage administration does not improve wound healing in wild type mice

**Figure 3:**
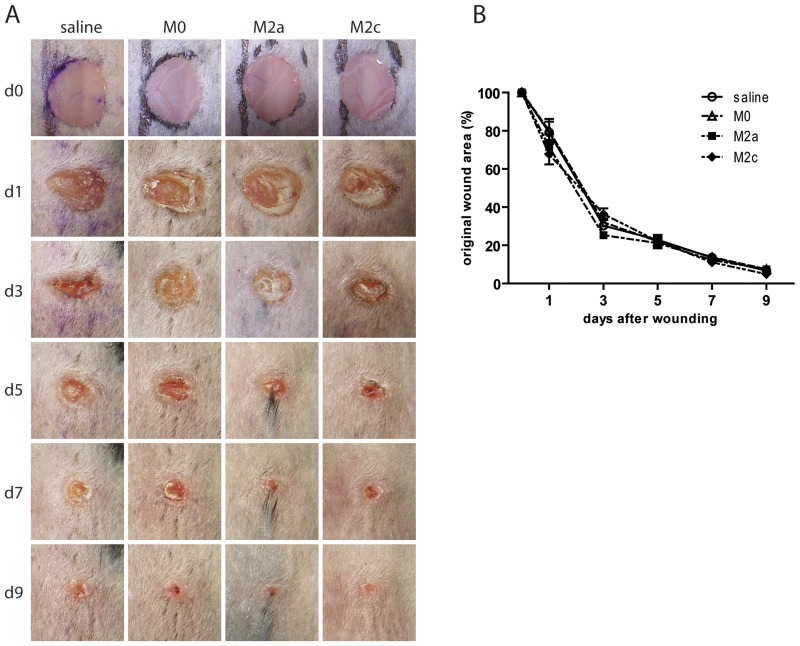
Wound healing in M2 macrophage injected wounds generated in C57BL/6 mice. (A) Macroscopic appearance of representative saline, M0, M2a, and M2c injected wounds. Day 0 pictures were taken immediately after wounding. (B) Quantification of wound closure rate. At the indicated days, wound areas were determined using image analysis and expressed as percentage of wound area immediately post-injury as described in methods (n = 6 mice/group). Statistical significance was evaluated with a two-way ANOVA with Bonferroni post-test.

Four groups of C57BL/6 mice were wounded on the dorsal surface as described in the method section, and cell suspensions of either M2-polarized, M0 (non-polarized) macrophages or saline were injected intradermally around the wound at three injection sites 1 and 3 days after wounding and wound closure was monitored every 2 days. In agreement with other studies [23], the cutaneous wound healing response progressed very rapidly in wild type mice with average wound area decreasing approximately 30% after 1 day and up to 60–70% after 3 days from injury. Wounds were almost completely closed 9 days after wounding. However, as shown in Fig 3A and B, no significant improvement could be measured in M2 macrophage treated wounds compared to M0 macrophage or saline treated wounds.

### 3.4. M2-polarized macrophage administration delays wound healing in diabetic mice

**Figure 4:**
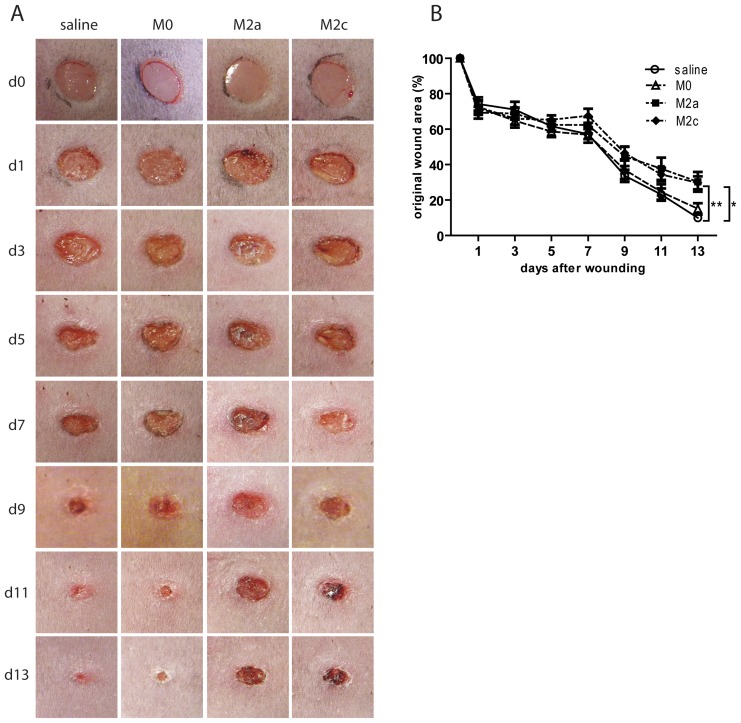
Wound healing in M2 macrophage injected wounds generated in diabetic db/db mice. (A) Macroscopic appearance of representative saline, M0, M2a, and M2c injected wounds. Day 0 pictures were taken immediately after wounding. (B) Quantification of wound closure rate. At the indicated days, wound areas were determined using image analysis and expressed as percentage of wound area immediately post-injury as described in methods (n = 6 mice/group). Statistical significance was evaluated with a two-way ANOVA with Bonferroni post-test. * p<0.05 M0 vs. M2a and M0 vs. M2c. ** p<0.01 saline vs. M2a and saline vs. M2c.

Since wild type mice heal wounds very rapidly and for the most part by wound contraction, we performed experiments in hyperglycemic db/db mice, which bear a spontaneous mutation in the leptin receptor gene and represent a model for human obesity and type 2 diabetes. Hyperglycemic db/db mice become obese shortly after birth and exhibit delayed wound healing, requiring approximately twice as much time for wound closure compared with non-diabetic mice [24]. Moreover, different studies have suggested that defective healing of diabetic wounds is due, at least partly, to macrophage dysfunction [13] and their impaired transition from pro-inflammatory to pro-healing phenotypes [14]. Therefore, we performed the wound healing experiment in db/db mice in the same setting as described for wild-type mice and monitored wound closure over time. As shown in Fig. 4, and in agreement with other studies [23], 4 mm wounds heal in approximately 15 days in db/db mice. Quantification of wound area indicated no significant differences in the healing pattern in the different groups until day 7 post-injury. However, from approximately day 7, wounds of M2a and M2c injected mice showed a decreased capacity to heal and remained significantly larger until day 13 (average % wound area ± SEM of saline: 9.93±1.15; M0: 15.0±3.18; M2a: 30.3±5.57; M2c: 29.7±3.68) (Fig. 4A and B).

### 3.5. M2 macrophages retain the M2 polarization profile 15 days post-injury

**Figure 5:**
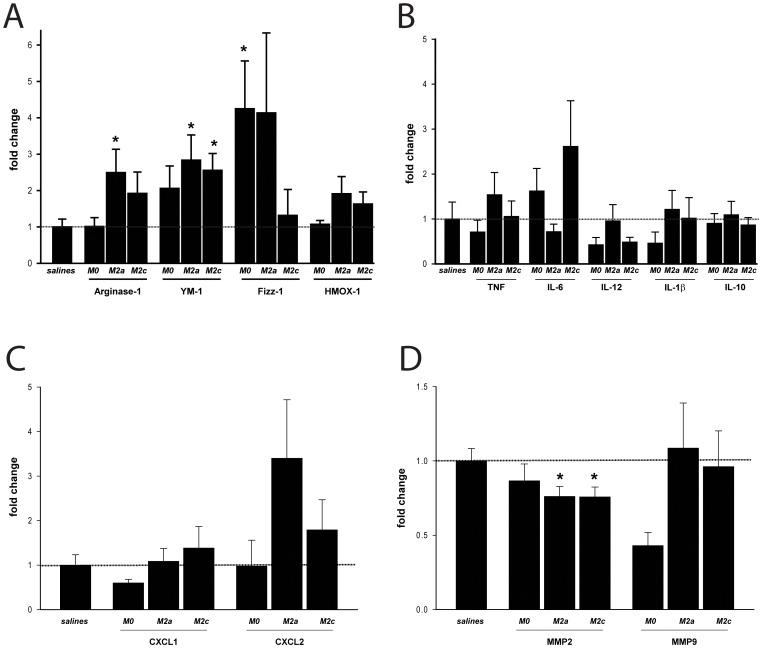
Gene expression analysis of db/db wounds isolated at day 15 post-wounding. Wounds were isolated from db/db mice 15 days after wounding and analyzed by real-time PCR for expression levels of (A) macrophage M2 markers Arginase-1, YM-1, Fizz-1 and HMOX-1; (B) inflammatory markers TNF, IL-6, IL-12, IL-1β and IL-10; (C) chemokines CXCL1 and CXCL2 and (D) matrix metalloproteinases MMP2 and MMP9. Statistical significance was evaluated analyzing M0, M2a and M2c expression levels compared to salines by t-test. *p<0.05. n = 6 mice (wounds)/group.

At 15 days post-injury, mice were sacrificed and wounds were isolated for analysis. Real time PCR analysis on RNA extracted from wound tissue indicated that M2 polarization markers like Arginase-1, YM-1 and Fizz-1 were significantly up regulated in M2 macrophage injected wounds compared to salines (Fig. 5A). Differentiation of macrophages from bone marrow stem cells requires the presence in the macrophage culture medium of 15% L-929 cell-conditioned medium (LCM) containing M-CSF. Since it was shown that M-CSF treated macrophages exhibit anti-inflammatory phenotypes [25], it is not surprising that M0 macrophage treated wounds are also exhibiting some traits of M2 differentiation with increased expression of Fizz-1 (Fig. 5A). Analysis of inflammatory cytokines indicated non-significant differences between the groups at day 15 post-injury (Fig. 5B), however at this time point, peak inflammatory responses have largely subsided. Chemokine analysis indicated that while CXCL1 levels did not vary between groups, levels of CXCL2 were increased in M2a and M2c wounds, although differences did not reach statistical significance (Fig. 5C). Tissue repair is also strongly influenced by the expression of matrix metalloproteinases (MMPs) due to the ability of these enzymes to remodel extracellular matrix components. Since we found reduced expression of MMP2 in M2a and M2c polarized macrophages and reduced MMP9 levels in M2c cells in-vitro (Fig. S1), we have examined the expression of these MMPs in-vivo, in day 15 wounds. MMP2 levels were significantly lower in M2a and M2c wounds compared to salines, confirming in-vitro data, while MMP9 levels did not show differences (Fig. 5D).

### 3.6. Histological analysis of wounds at day 15 post-injury

**Figure 6:**
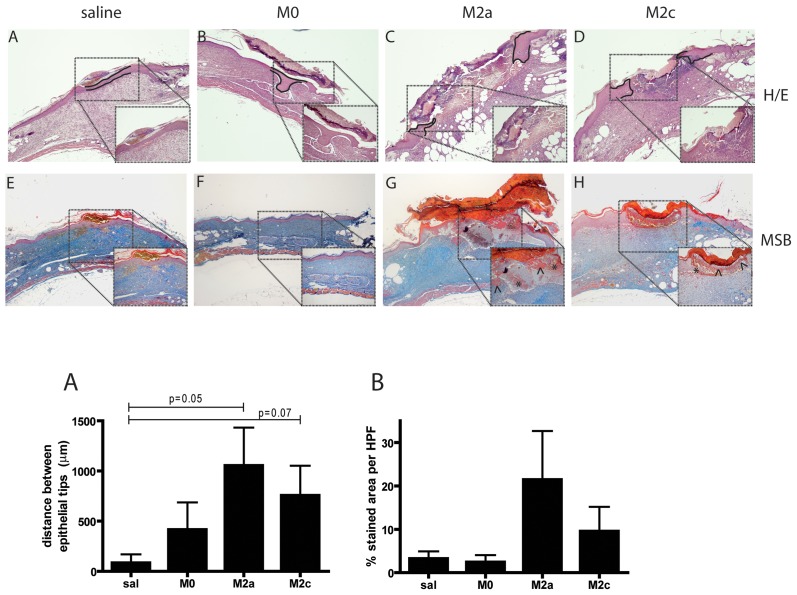
Histological analysis of db/db wounds isolated at day 15 post-wounding. Wounds from saline or macrophage injected db/db mice were isolated 15 days after wounding and processed for generation of paraffin sections. (A-D) H&E stained sections indicating migrating epithelial tongues (dotted line) in M2a and M2c wounds while complete re-epithelialization is present in saline and M0 wounds. (E-H) MSB trichrome staining indicating increased presence of fibrin (marked by arrowheads) and erythrocytes (marked by stars) in M2 macrophage injected wounds. Magnification 40x with 100x insets. Graphs are quantifications of (A) re-epithelialization and (B) fibrin/erythrocyte staining respectively. Statistical significance was evaluated analyzing M0, M2a and M2c expression levels compared to salines by t-test. n = 6 mice (wounds)/group.

Histological examination of H&E stained wounds isolated at day 15 post-injury (Fig. 6A-D) indicated that M2 macrophage injected wounds presented a delayed healing phenotype with incomplete re-epithelialization and presence of numerous erythrocytes at the wound bed. To quantify the degree of re-epithelialization, the distance between the tips of the migrating epithelial tongues was measured (graph; Fig. 6A). Re-epithelialization was significantly reduced in M2a wounds compared to controls (p = 0.050). The same trend was observed for the M2c group, though differences were borderline non-significant (p = 0.073). To obtain a quantitative evaluation of vessel permeability resulting in the leakage of plasma components such as fibrinogen and erythrocytes in the extracellular compartment, an MSB trichrome staining was performed, in which fibrin and erythrocytes stain red/orange and connective tissue blue (Fig. 6E-H). M2 macrophage injected wounds showed a trend towards increased fibrin deposition and presence of erythrocytes in the wound bed, indicative of increased vessel leakage, and suggesting that these wounds might be at an earlier stage of healing compared to salines and M0 controls (graph; Fig 6B).

**Figure 7:**
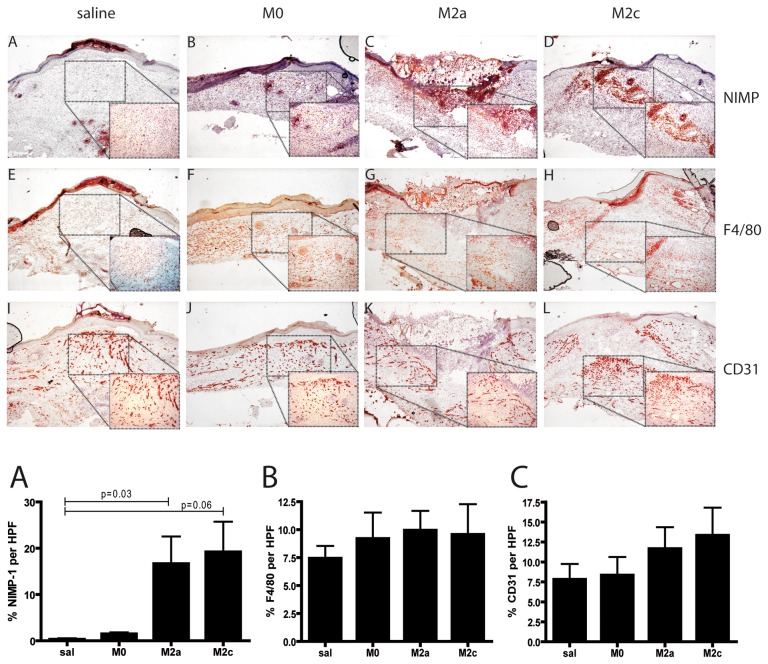
Immunohistochemical analysis of db/db wounds isolated at day 15 post-wounding. Immunohistochemical stainings were performed in cryosections. (A-D) NIMP staining for wound neutrophils indicated higher numbers of neutrophils in M2a and M2c treated wounds compared to saline and M0 groups. Mice analyzed per group: salines = 3; M0 = 5; M2a = 6; M2c = 5. (E-H) F4/80 staining for macrophages indicated not significant differences between the groups. Mice analyzed per group: salines = 4; M0 = 4; M2a = 6; M2c = 6. (I-L) CD31 staining indicated similar staining for endothelial cells in the different groups. Mice analyzed per group: salines = 4; M0 = 5; M2a = 5; M2c = 4. Magnification 40x with 100x insets. Graphs are quantifications of (A) NIMP, (B) F4/80 and (C) angiogenesis staining respectively. Statistical significance was evaluated analyzing M0, M2a and M2c expression levels compared to salines by t-test.

In order to evaluate collagen fiber density and maturation in the granulation tissue of wounds, picrosirius red staining was performed and viewed under polarized light. Contrarily to saline wounds that showed intense, thick and continuous bands of birefringence, M2a and M2c treated wounds showed modest birefringence intensity which reflected short and immature collagen fiber bundles arranged in a random pattern (Fig. S2). Since the collagen fiber bundles in granulation tissue become more organized as the connective tissue in wounds matures, the birefringence pattern in M2a and M2c wounds reflects a young, immature granulation tissue. To further characterize wound phenotype, immunohistochemical stainings for quantification of macrophages, neutrophils and the extent of neovascularization were performed. As shown in Fig. 7, quantification of F4/80 (graph 7B) and CD31 (graph 7C) stainings indicated comparable amount of macrophages and angiogenesis between the groups. However, M2 macrophage injected wounds showed persistence of neutrophils (graph 7A; p = 0.037 M2a vs. salines and p = 0.061 M2c vs. salines) in the wound bed 15 days post-injury compared to saline and M0 macrophage injected wounds.

Taken together, M2 macrophage injected wounds presented a phenotype of delayed healing with delayed re-epithelialization, increased vessel leakage, more immature granulation tissue and persistence of neutrophils at late stages of repair.

## Discussion

The role of macrophages during cutaneous wound healing responses has been the subject of intense research over the past decades. By making use of macrophage depleted mouse models, recent studies have established an essential role for these cells in wound repair [6]–[8]. From these studies it appeared that macrophage ablation results in delayed wound closure that is associated with features such as delayed re-epithelialization, reduced wound contraction due to impaired myofibroblast differentiation, reduced angiogenesis associated with increased endothelial cell apoptosis and reduced granulation tissue formation. In addition it was shown that, among the function of macrophages during the inflammatory phase of repair is the promotion of macrophage alternative activation that will consecutively support angiogenesis and myofibroblast differentiation [26]. However, if on one hand macrophage deficiency is detrimental for wound healing, uncontrolled macrophage activation can result in derailed healing and chronic wounds [12], [15].

Adoptive transfer of in-vitro derived alternative activated M2 macrophages was proven beneficial in a number of chronic inflammatory diseases in mice [17]–[21]. Moreover, we have recently shown that M2 macrophages have both pro-angiogenic and pro-arteriogenic potential in both in-vitro and in-vivo mouse models [27], [28]. Hence, in this study we tested the hypothesis that administration of M2-polarized macrophages in wounds during the early inflammatory phase, might improve cutaneous wound healing responses by influencing different processes in the wound microenvironment like anti-inflammatory phenotypes, resolution of inflammation and promotion of angiogenesis. Unexpectedly we observed no improvement in wound healing rate in C57BL/6 mice by administration of M2 macrophages while wound closure was even delayed in genetically diabetic db/db mice. Using eGFP expressing macrophages we could trace exogenously administered cells in wounds until approximately day 8 post-injection. Moreover, gene expression analysis of day 15 wounds showed increased expression of alternative activation markers such as Arginase-1 and YM-1 in M2 wounds, indicating persistence of M2 polarization phenotype even after 15 days of macrophage injection. Although expression of cytokines between the different groups did not differ at this time point, M2 injected wounds presented a significant reduction in expression of MMP2 compared to controls. MMPs have been implicated in wound repair by their ability to remodel extracellular matrix components and indiscriminate pharmacologic inhibition of MMPs significantly delays wound healing in wild type mice, indicating their substantial role in skin repair and re-epithelialization [29], [30]. A reduction in MMPs levels might therefore contribute to the phenotype of delayed healing in M2a and M2c wounds in our experiment. Reduced expression of MMP2 in M2a and M2c wounds could be a consequence of its reduced expression by M2a and M2c wound macrophages, as our in-vitro analysis of M2a and M2c cells has indicated. Our observation that murine M2 macrophages express reduced MMP2 levels is supported by studies in human macrophages where alternative activation was found to decrease mRNA levels and gelatinase activity of MMP2 [31].

Microscopic examination of wounds in the diabetic mice indicated that M2 macrophage injected wounds presented a phenotype of delayed re-epithelialization, with a younger and more immature connective tissue as reflected by collagen birefringence under polarized light, and with persistence of neutrophils in the wound bed. Neutrophils negatively influence wound repair probably because this cell type is capable of destroying normal tissue. Their depletion was previously shown to accelerate wound healing in both wild-type and diabetic mice [32], and delayed wound closure in diabetic mice has been attributed to prolonged persistence of neutrophils and macrophages in the late phases of repair [33]. Although it is still not clear if increased neutrophil numbers in M2 injected wounds in our experiments are due to late infiltration of these cells or to defective resolution of inflammation and neutrophil clearance, we can hypothesize that neutrophil persistence retards the healing process of these wounds.

While M2a and M2c wounds presented comparable phenotypes, slight differences in wound gene expression, re-epithelialization and erythrocyte/fibrin content were found, indicating that polarized macrophages maintain distinct functional phenotypes in-vivo.

Our studies are in line with a recent report by Dreymueller et al. [34], in which embryonic stem-cell derived macrophages (ESDM), that exhibit an M2-like phenotype, were shown to prolong the healing of deep skin wounds generated by removal of a skin flap in the tail base of mice. Similarly to the diabetic db/db mice we used in our study, this model allows for an extended healing time and avoids the rapid wound closure by contraction as occurs in wild-type mice [35]. Comparable to our findings, ESDM treated wounds presented reduced re-epithelialization and significantly higher cellularity compared to controls, indicating persistence of inflammation during the late phases of repair.

It can be hypothesized that M2 macrophages might switch towards a more M1-like phenotype upon injection to the early wounds which exhibit a pro-inflammatory milieu. However, the discrepancy in phenotype between M2 and the control M0 injected wounds would argue against a reverse polarization of exogenously administered cells by the wound environment. Conversely we can speculate that exogenously administered M2 cells, but not the non-polarized M0 controls, might induce the M1 macrophages present in the wounds at the initial phases of repair, to adopt a less inflammatory profile that ultimately negatively affects healing. Injection of activated macrophages into wounds of patients was shown to promote wound healing [36], while rapid recruitment and activation of macrophages into murine wounds accelerates tissue repair [37]. In line, ablation of macrophages during the early phases of repair [6] or loss of TNF [38], was shown to retard re-epithelialization and wound closure of murine cutaneous wounds. Therefore it can be hypothesized that some degree of initial pro-inflammatory response is not damaging, but on the contrary, necessary in order to activate the subsequent phases of healing, and is the unrestrained, prolonged or excessive activation of inflammation that is detrimental. The administration of M2 macrophages during the early inflammatory phases in our experiments might have disrupted this delicate balance. The potential beneficial effect of M2 macrophage administration in later phases of healing needs to be evaluated.

Taken together, our study shows that, at least during murine cutaneous healing responses, manipulation of the wound environment by exogenous administration of M2-polarized macrophages does not represent an effective therapeutic strategy. Alternative ways to modify the wound phenotype into a less-inflammatory, pro-healing profile, like blocking pro-inflammatory factors as IL-1β [39] or TNF [15], [40] have proven more promising therapeutic approaches.

## Supporting Information

Figure S1 **MMP2 and MMP9 expression analysis in M0, M2a and M2c macrophages.** Cells were analyzed in quadruplicate samples after 24 h of polarization. Statistical significance was evaluated analyzing M2a and M2c expression levels compared to M0, by t-test. **p<0.01.
(TIF)

Figure S2 **Representative images of picrosirius red (collagen deposition) stained wounds.** Picrosirius red stained sections were analyzed under bright field light (A-D) or under polarized light (E-H). Intense red birefringence seen in saline and M0 wounds is a consequence of uniform packing of newly deposited collagen fiber bundles that are arranged in parallel arrays while minimal organization of collagen bundles results in little red birefringence intensity as seen in M2a and M2c wounds. Magnification 100x.
(TIF)

Table S1 **qPCR primer sequences used in this study.**
(DOC)

## References

[1] Eming SA, Krieg T, Davidson JM (2007) Inflammation in wound repair: molecular and cellular mechanisms. J Invest Dermatol 127: 514–525. 17299434 10.1038/sj.jid.5700701

[2] Gurtner GC, Werner S, Barrandon Y, Longaker MT (2008) Wound repair and regeneration. Nature 453: 314–321. 18480812 10.1038/nature07039

[3] DiPietro LA, Polverini PJ, Rahbe SM, Kovacs EJ (1995) Modulation of JE/MCP-1 expression in dermal wound repair. Am J Pathol 146: 868–875. 7717454 PMC1869244

[4] Swirski FK, Nahrendorf M, Etzrodt M, Wildgruber M, Cortez-Retamozo V, et al (2009) Identification of splenic reservoir monocytes and their deployment to inflammatory sites. Science 325: 612–616. 19644120 10.1126/science.1175202 PMC2803111

[5] MacDonald KP, Palmer JS, Cronau S, Seppanen E, Olver S, et al (2010) An antibody against the colony-stimulating factor 1 receptor depletes the resident subset of monocytes and tissue- and tumor-associated macrophages but does not inhibit inflammation. Blood 116: 3955–3963. 20682855 10.1182/blood-2010-02-266296

[6] Lucas T, Waisman A, Ranjan R, Roes J, Krieg T, et al (2010) Differential roles of macrophages in diverse phases of skin repair. J Immunol 184: 3964–3977. 20176743 10.4049/jimmunol.0903356

[7] Goren I, Allmann N, Yogev N, Schurmann C, Linke A, et al (2009) A transgenic mouse model of inducible macrophage depletion: effects of diphtheria toxin-driven lysozyme M-specific cell lineage ablation on wound inflammatory, angiogenic, and contractive processes. Am J Pathol 175: 132–147. 19528348 10.2353/ajpath.2009.081002 PMC2708801

[8] Mirza R, DiPietro LA, Koh TJ (2009) Selective and specific macrophage ablation is detrimental to wound healing in mice. Am J Pathol 175: 2454–2462. 19850888 10.2353/ajpath.2009.090248 PMC2789630

[9] Mantovani A, Sica A, Sozzani S, Allavena P, Vecchi A, et al (2004) The chemokine system in diverse forms of macrophage activation and polarization. Trends Immunol 25: 677–686. 15530839 10.1016/j.it.2004.09.015

[10] Mosser DM, Edwards JP (2008) Exploring the full spectrum of macrophage activation. Nat Rev Immunol 8: 958–969. 19029990 10.1038/nri2448 PMC2724991

[11] Daley JM, Brancato SK, Thomay AA, Reichner JS, Albina JE (2010) The phenotype of murine wound macrophages. J Leukoc Biol 87: 59–67. 20052800 10.1189/jlb.0409236 PMC2801619

[12] Sindrilaru A, Peters T, Wieschalka S, Baican C, Baican A, et al (2011) An unrestrained proinflammatory M1 macrophage population induced by iron impairs wound healing in humans and mice. J Clin Invest 121: 985–997. 21317534 10.1172/JCI44490 PMC3049372

[13] Khanna S, Biswas S, Shang YL, Collard E, Azad A, et al. (2010) Macrophage Dysfunction Impairs Resolution of Inflammation in the Wounds of Diabetic Mice. Plos One 5.. 10.1371/journal.pone.0009539 PMC2832020 20209061

[14] Mirza R, Koh TJ (2011) Dysregulation of monocyte/macrophage phenotype in wounds of diabetic mice. Cytokine 56: 256–264. 21803601 10.1016/j.cyto.2011.06.016

[15] Goren I, Muller E (2007) Schiefelbein D, Christen U, Pfeilschifter J, et al (2007) Systemic anti-TNFalpha treatment restores diabetes-impaired skin repair in ob/ob mice by inactivation of macrophages. J Invest Dermatol 127: 2259–2267. 17460730 10.1038/sj.jid.5700842

[16] Lai JJ, Lai KP, Chuang KH, Chang P, Yu IC, et al (2009) Monocyte/macrophage androgen receptor suppresses cutaneous wound healing in mice by enhancing local TNF-alpha expression. J Clin Invest 119: 3739–3751. 19907077 10.1172/JCI39335 PMC2786793

[17] Hunter MM, Wang A, Parhar KS, Johnston MJ, Van Rooijen N, et al (2010) In vitro-derived alternatively activated macrophages reduce colonic inflammation in mice. Gastroenterology 138: 1395–1405. 20044996 10.1053/j.gastro.2009.12.041

[18] Parsa R, Andresen P, Gillett A, Mia S, Zhang XM, et al (2012) Adoptive transfer of immunomodulatory M2 macrophages prevents type 1 diabetes in NOD mice. Diabetes 61: 2881–2892. 22745325 10.2337/db11-1635 PMC3478537

[19] Wang Y, Wang YP, Zheng G, Lee VW, Ouyang L, et al (2007) Ex vivo programmed macrophages ameliorate experimental chronic inflammatory renal disease. Kidney Int 72: 290–299. 17440493 10.1038/sj.ki.5002275

[20] Weber MS, Prod'homme T, Youssef S, Dunn SE, Rundle CD, et al (2007) Type II monocytes modulate T cell-mediated central nervous system autoimmune disease. Nat Med 13: 935–943. 17676050 10.1038/nm1620

[21] Li K, Xu W, Guo Q, Jiang Z, Wang P, et al (2009) Differential macrophage polarization in male and female BALB/c mice infected with coxsackievirus B3 defines susceptibility to viral myocarditis. Circ Res 105: 353–364. 19608981 10.1161/CIRCRESAHA.109.195230

[22] Giel-Moloney M, Krause DS, Chen G, Van Etten RA, Leiter AB (2007) Ubiquitous and uniform in vivo fluorescence in ROSA26-EGFP BAC transgenic mice. Genesis 45: 83–89. 17269129 10.1002/dvg.20269 PMC2121618

[23] Grochot-Przeczek A, Lach R, Mis J, Skrzypek K, Gozdecka M, et al (2009) Heme oxygenase-1 accelerates cutaneous wound healing in mice. PLoS One 4: e5803. 19495412 10.1371/journal.pone.0005803 PMC2686151

[24] Greenhalgh DG, Sprugel KH, Murray MJ, Ross R (1990) PDGF and FGF stimulate wound healing in the genetically diabetic mouse. Am J Pathol 136: 1235–1246. 2356856 PMC1877595

[25] Verreck FA, de Boer T, Langenberg DM, Hoeve MA, Kramer M, et al (2004) Human IL-23-producing type 1 macrophages promote but IL-10-producing type 2 macrophages subvert immunity to (myco)bacteria. Proc Natl Acad Sci U S A 101: 4560–4565. 15070757 10.1073/pnas.0400983101 PMC384786

[26] Brancato SK, Albina JE (2011) Wound macrophages as key regulators of repair: origin, phenotype, and function. Am J Pathol 178: 19–25. 21224038 10.1016/j.ajpath.2010.08.003 PMC3069845

[27] Jetten N, Donners MM, Wagenaar A, Cleutjens JP, van Rooijen N, et al (2013) Local delivery of polarized macrophages improves reperfusion recovery in a mouse hind limb ischemia model. PLoS One 8: e68811. 23894348 10.1371/journal.pone.0068811 PMC3722193

[28] Jetten N, Verbruggen S, Gijbels MJ, Post MJ, De Winther MP, et al (2014) Anti-inflammatory M2, but not pro-inflammatory M1 macrophages promote angiogenesis in vivo. Angiogenesis 17: 109–118. 24013945 10.1007/s10456-013-9381-6

[29] Agren MS, Mirastschijski U, Karlsmark T, Saarialho-Kere UK (2001) Topical synthetic inhibitor of matrix metalloproteinases delays epidermal regeneration of human wounds. Exp Dermatol 10: 337–348. 11589731 10.1034/j.1600-0625.2001.100506.x

[30] Mirastschijski U, Impola U, Karsdal MA, Saarialho-Kere U, Agren MS (2002) Matrix metalloproteinase inhibitor BB-3103 unlike the serine proteinase inhibitor aprotinin abrogates epidermal healing of human skin wounds ex vivo. J Invest Dermatol 118: 55–64. 11851876 10.1046/j.0022-202x.2001.01652.x

[31] Huang WC, Sala-Newby GB, Susana A, Johnson JL, Newby AC (2012) Classical macrophage activation up-regulates several matrix metalloproteinases through mitogen activated protein kinases and nuclear factor-kappaB. PLoS One 7: e42507. 22880008 10.1371/journal.pone.0042507 PMC3411745

[32] Dovi JV, He LK, DiPietro LA (2003) Accelerated wound closure in neutrophil-depleted mice. J Leukoc Biol 73: 448–455. 12660219 10.1189/jlb.0802406

[33] Wetzler C, Kampfer H, Stallmeyer B, Pfeilschifter J, Frank S (2000) Large and sustained induction of chemokines during impaired wound healing in the genetically diabetic mouse: prolonged persistence of neutrophils and macrophages during the late phase of repair. J Invest Dermatol 115: 245–253. 10951242 10.1046/j.1523-1747.2000.00029.x

[34] Dreymueller D, Denecke B, Ludwig A, Jahnen-Dechent W (2013) Embryonic stem cell-derived M2-like macrophages delay cutaneous wound healing. Wound Repair Regen 21: 44–54. 23126541 10.1111/j.1524-475X.2012.00858.x

[35] Falanga V, Schrayer D, Cha J, Butmarc J, Carson P, et al (2004) Full-thickness wounding of the mouse tail as a model for delayed wound healing: accelerated wound closure in Smad3 knock-out mice. Wound Repair Regen 12: 320–326. 15225210 10.1111/j.1067-1927.2004.012316.x

[36] Danon D, Madjar J, Edinov E, Knyszynski A, Brill S, et al (1997) Treatment of human ulcers by application of macrophages prepared from a blood unit. Exp Gerontol 32: 633–641. 9785089 10.1016/s0531-5565(97)00094-6

[37] Wigglesworth KM, Racki WJ, Mishra R, Szomolanyi-Tsuda E, Greiner DL, et al (2011) Rapid recruitment and activation of macrophages by anti-Gal/alpha-Gal liposome interaction accelerates wound healing. J Immunol 186: 4422–4432. 21357545 10.4049/jimmunol.1002324 PMC4091898

[38] Shinozaki M, Okada Y, Kitano A, Ikeda K, Saika S (2009) Impaired cutaneous wound healing with excess granulation tissue formation in TNFalpha-null mice. Arch Dermatol Res 301: 531–537. 19543902 10.1007/s00403-009-0969-z

[39] Mirza RE, Fang MM, Ennis WJ, Koh TJ (2013) Blocking interleukin-1beta induces a healing-associated wound macrophage phenotype and improves healing in type 2 diabetes. Diabetes 62: 2579–2587. 23493576 10.2337/db12-1450 PMC3712034

[40] Ashcroft GS, Jeong MJ, Ashworth JJ, Hardman M, Jin W, et al (2012) Tumor necrosis factor-alpha (TNF-alpha) is a therapeutic target for impaired cutaneous wound healing. Wound Repair Regen 20: 38–49. 22151742 10.1111/j.1524-475X.2011.00748.x PMC3287056

