# Supplementary figures and images for: Wound Administration of M2-Polarized Macrophages Does Not Improve Murine Cutaneous Healing Responses
Source: PLoS One. 2014 Jul 28;9(7):e102994. doi: 10.1371/journal.pone.0102994 (PMC4113363; doi:10.1371/journal.pone.0102994)

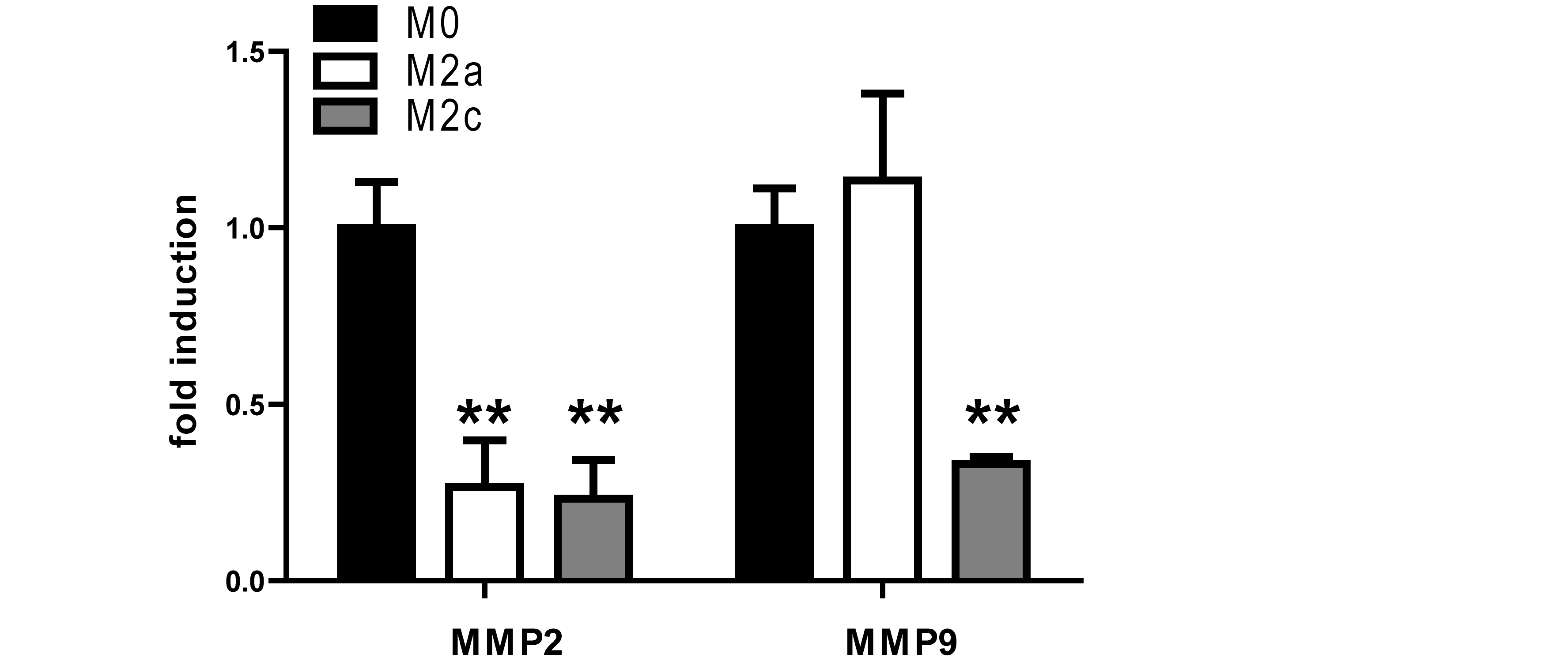

Supplement: Figure S1 — MMP2 and MMP9 expression analysis in M0, M2a and M2c macrophages. Cells were analyzed in quadruplicate samples after 24 h of polarization. Statistical significance was evaluated analyzing M2a and M2c expression levels compared to M0, by t-test. **p<0.01. (TIF) [file pone.0102994.s001.tif]

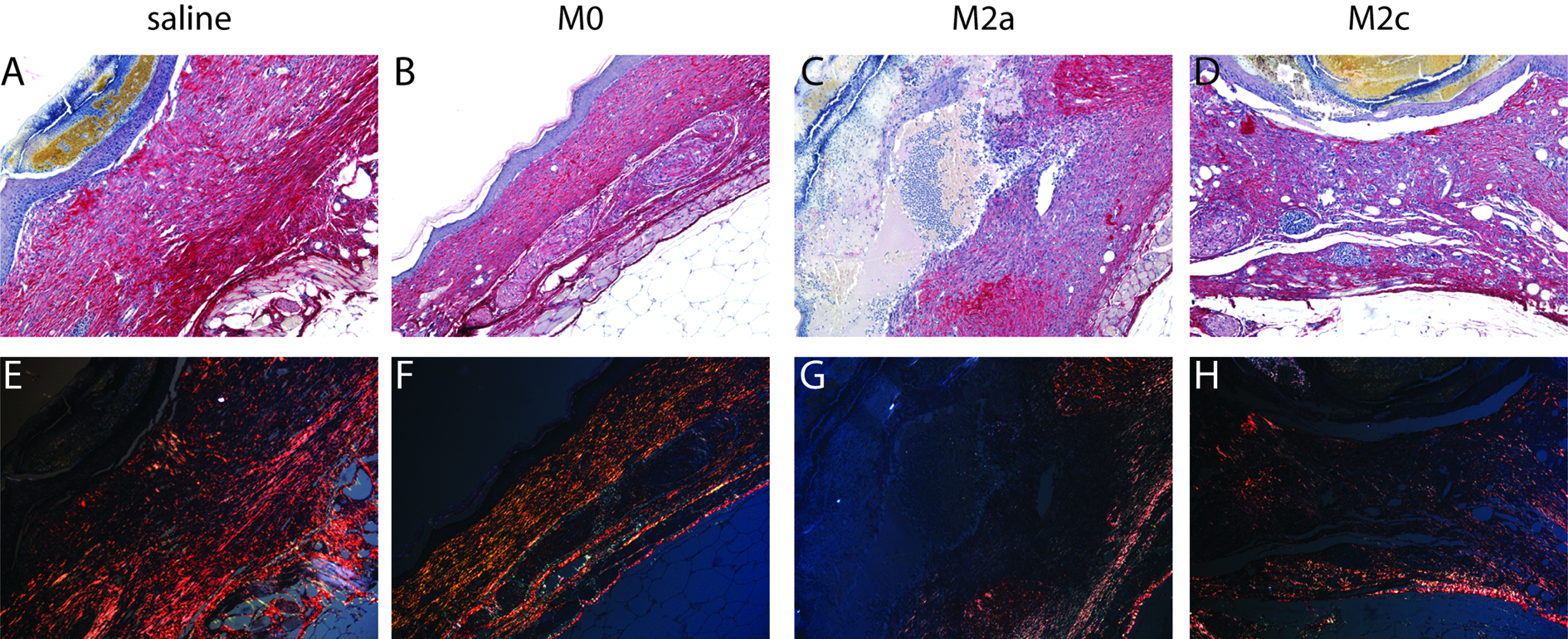

Supplement: Figure S2 — Representative images of picrosirius red (collagen deposition) stained wounds. Picrosirius red stained sections were analyzed under bright field light (A-D) or under polarized light (E-H). Intense red birefringence seen in saline and M0 wounds is a consequence of uniform packing of newly deposited collagen fiber bundles that are arranged in parallel arrays while minimal organization of collagen bundles results in little red birefringence intensity as seen in M2a and M2c wounds. Magnification 100x. (TIF) [file pone.0102994.s002.tif]
