# Supplementary material for: Wound Administration of M2-Polarized Macrophages Does Not Improve Murine Cutaneous Healing Responses
Source: PLoS One. 2014 Jul 28;9(7):e102994. doi: 10.1371/journal.pone.0102994 (PMC4113363; doi:10.1371/journal.pone.0102994)
Supplement: Table S1 — qPCR primer sequences used in this study. (DOC) [file pone.0102994.s003.doc]

| Gene | Forward primer (5’ 🡪 3’) | Reverse primer (5’ 🡪 3’) |
| --- | --- | --- |
| *Arg1* | CATGGGCAACCTGTGTCCTT | CGATGTCTTTGGCAGATATGCA |
| *Ym1* | TGGCCCACCAGGAAAGTACA | CAGTGGCTCCTTCATTCAGAAA |
| *Fizz1* | GGAACTTCTTGCCAATCCAG | ACACCCAGTAGCAGTCATCCC |
| *Hmox1* | CCGCCTTCCTGCTCAACAT | ATCTGTGAGGGACTCTGGTCTTTG |
| *TNF* | CATCTTCTCAAAATTCGAGTGACAA | TGGGAGTAGACAAGGTACAACCC |
| *IκBα* | TGGAAGTCATTGGTCAGGTGAA | CAGAAGTGCCTCAGCAATTCCT |
| *IL-6* | TTCAACCAAGAGGTAAAAGATTTACATAA | CACTCCTTCTGTGACTCCAGCTT |
| *IL-1β* | AAAGAATCTATACCTGTCCTGTGTAATGAAA | GGTATTGCTTGGGATCCACACT |
| *IL-12* | TGAGAACTACAGCACCAGCTTCTT | CTTCAAAGGCTTCATCTGCAAGT |
| *IL-10* | GCTCTTACTGACTGGCATGAG | CGCAGCTCTAGGAGCATGTG |
| *CXCL1* | CAAAAGATGCTAAAAGGTGTCCCCA | CGTTCACCAGACAGGTGCCAT |
| *CXCL2* | CCCTGGTTCAGAAAATCATCCAAA | TTTGGTTCTTCCGTTGAGGGAC |
| *MMP2* | CGATGTCGCCCCTAAAACAG | CTTGAGGGTATCTTTCAGCACAAA |
| *MMP9* | TCTTCCCCAAAGACCTGAAAAC | GCCCGGGTGTAACCATAGC |
| *cyclophilin A* | TTCCTCCTTTCACAGAATTATTCCA | CCGCCAGTGCCATTATGG |
| *β-actin* | GACAGGATGCAGAAGGAGATTACTG | CCACCGATCCACACAGAGTACTT |
|  |  |  |
|  |  |  |
|  |  |  |
|  |  |  |
|  |  |  |
|  |  |  |
|  |  |  |
|  |  |  |
|  |  |  |
|  |  |  |
|  |  |  |
|  |  |  |
|  |  |  |
|  |  |  |
|  |  |  |
|  |  |  |

Supplementary Table 1: qPCR primer sequences used in this study
